# Clinical outcome observation of the embolization of orbital vascular malformation with medical glue under direct intra-operative view

**DOI:** 10.1186/s12886-018-1002-0

**Published:** 2018-12-20

**Authors:** Tingting Lin, Limin Zhu, Yanjin He

**Affiliations:** Tianjin Medical University Eye Hospital, School of Optometry and Ophthalmology, TMU, Tianjin Medical University Eye Institute, No.251 Fu Kang Road, Nankai District, Tianjin, 300384 People’s Republic of China

**Keywords:** Orbit, Vascular malformation, Medical glue, Embolization

## Abstract

**Background:**

Orbital vascular malformation often encircles normal tissue with ill-defined borders. It is easy to bleed during resection operation, making surgical treatment difficult and lesions hard to be removed completely. In this study we aimed to summarize the treatment outcomes by embolizing orbital vascular malformation with intraoperative intracavitary injection of medical glue .

**Methods:**

A retrospective observational and cross-sectional case series study enrolled 31 patients (male = 9, female = 22) with orbital vascular malformations, who were treated from March 2008 to September 2017 at our institution. The clinical features, operation records, pathological reports and follow-up data were analyzed.

**Results:**

The location of vascular malformations involved intraorbital (14 cases), superficial area of eyelid and/or face (7 cases), both intraorbital and superficial area (10 cases). Imaging examination showed a solitary mass with regular shape in 8 cases and a space occupying lesion with irregular shape and ill-defined margins in 23 cases. There were 9 cases had optic nerve involved. Surgical debulkling were performed via skin incision on the mass surface (5 cases), lateral orbitotomy (2 cases), and anterior orbitotomy (24 cases). During the operation, lesions were partly exposed and injected with medical glue. The amount of injected glue was 0.25 ml to 2.5 ml in divided doses. The lesions and remnant glue were removed after the glue had turned hard. The whole procedure caused less bleeding and was easier performing than usual. Topical skin aseptic inflammation took place on the same side of the superficial eyelid lesions in 3 cases. One patient suffered from sudden central retinal artery embolism on the third day post operation. With timely rescue and appropriate procedure, visual acuity recovered to 20/32. There were no recurrences in 29 cases.

**Conclusions:**

Embolization of orbital vascular malformation with medical glue intraoperatively made it easy to control hemorrhage. Surgeons should be careful with glue application methods in order to avoid complications.

## Background

Orbital vascular malformation is a mass lesion made up of blood vessels. According to the origin of the blood vessel, the lesion can be categorized as venous, arterial and arterial-venous malformation. The malformed blood vessels often encircle the normal tissue in orbit and have ill-defined borders making surgical treatment of orbital vascular malformations difficult. Because of the risk of hemorrhage during operation, the malformed blood vessel may hard to be removed completely. Accordingly, there are many post-surgical complications and a high incidence of reoccurrence. In this article we summarized the method of embolization with medical glue during the surgical treatment of orbital vascular malformation as well as the resection of lesions, and the prognosis of patients.

## Methods

### Ethics approval and patient consent

This study was a retrospective, observational and cross-sectional case series and was approved by the Human Research Ethics Committee of the Tianjin Medical University Eye Hospital [No. 2017KY(L)-23] and complied with the Declaration of Helsinki. Patients with the orbital vascular malformation presented to TMUEH during Mar, 2008 to Sep, 2017 were enrolled. There were 31 cases (31 eyes) diagnosed as orbital vascular malformation and performed with the medical glue embolization under direct intra-operative view according to medical records. Written informed consents were obtained from the patients.

### Materials

The medical glue used is the EC ear-cephalic glue manufactured by Guangzhou Bai Yun medical glue company, of 1 ml standard (Type: GX812-EC).

### Data collection

The clinical characteristics, surgical records, pathological reports and follow-up records of 31 patients were reviewed retrospectively. The vascular malformation involved the right eye in 19 cases and the left eye in 12 cases. Patients included 9 males and 22 femalesd. The age of onset was from 2 to 67 years old and median age of 33 years.

## Results

### Clinical features

Table 1 shows the clinical character of the cases. Imaging examination showed an isolated lesion with regular shape in 8 cases, and an irregular soft tissue mass with ill-defined border in 23 cases, among which 9 cases had lesions surrounding or pressing the optic nerve.

**Table 1:** Clinical character of the cases

Clinical character	N
Cases	31
Gender(F/M)	22/9
Chief complaint	
Eyelid edema	6
Proptosis	9
Mass in their eyelids	16
Clinical manifestations	
Lesions were found at birth	4
Acute intra-orbital hemorrhage	4
Pain during onset of illness	8
Lesions changed with body position^a^	11
Pulsation and wind-blowing murmur	1
Locations of the involvements	
Both intra-orbital and superficial eyelid tissue	10
Inside the orbit	14
On the eyelids and face	7
Conjunctiva involved	4
Lesions shape showed on imaging examination	
Regular/irregular mass with ill-defined border	8/23
Size of lesions	
The largest/the smallest	30 mm ×20 mm × 20 mm/9 mm × 5 mm × 4 mm

^a^The lesions’ changes included the level of eyeball protrusion or the mass volume on imaging after body position changed

### Surgical method

Table 2 shows the therapeutic data of the cases.

**Table 2:** Therapeutic data of the cases

Therapeutic data	N
Anesthesia method	
Local anesthesia/general anesthesia	8/23
Surgical approach	
Routine lateral orbital bone incision	2
Skin incision on the surface of the lesion	5
Anterior orbitotomy approach	24
Skin incision under eyebrow	9
Skin incision under lower eyelid eyelash	4
Transconjunctiva joint lateral canthus incision	9
Transconjunctiva incision	2
Intra-orbital location of the vascular malformation	
Central−/extra-conal orbital compartment	12/12
The volume of injected medical glue	
0.25 ml/0.5 ml/0.75 ml/1 ml/1.5 ml/2.5 ml	3/10/1/11/5/1

During the operation, the intra-orbital soft tissue was carefully isolated to protect the normal structure, and the medical glue was injected into the mass when the anterior surface of the malformed blood vessel was exposed. In four cases the glue was diluted 1:1 with water for injection (WFI) before injection into the blood vessel. For the other 27 cases, undiluted glue was used. The volume of injected medical glue showed in Table 2. Specifically, 2.5 ml of glue was injected into the largest malformation in three separate injections; 0.25 ml was injected into the smallest malformation. About 10~15 s later the malformation was examined to determine whether it had been completely molded and solidified. If a soft malformation was still present locally, more injections were given. Nine cases were injected more than once, and two cases also used tungsten wire wrapped by gelatin sponge to fill the area. For the hemorrhage on the surface of the malformation, two cases also had medical glue applied to the surface of the lesion to stop bleeding. When the blood vessel was completely solidified, the surrounding soft tissue was isolated and the malformed blood vessel could be resected in one piece. In four cases because the lesion was next to the optic nerve, solidified blood vessel was only partially removed. Overall, hemorrhage during the operation was minimal.

### Pathological features

**Fig. 1:**
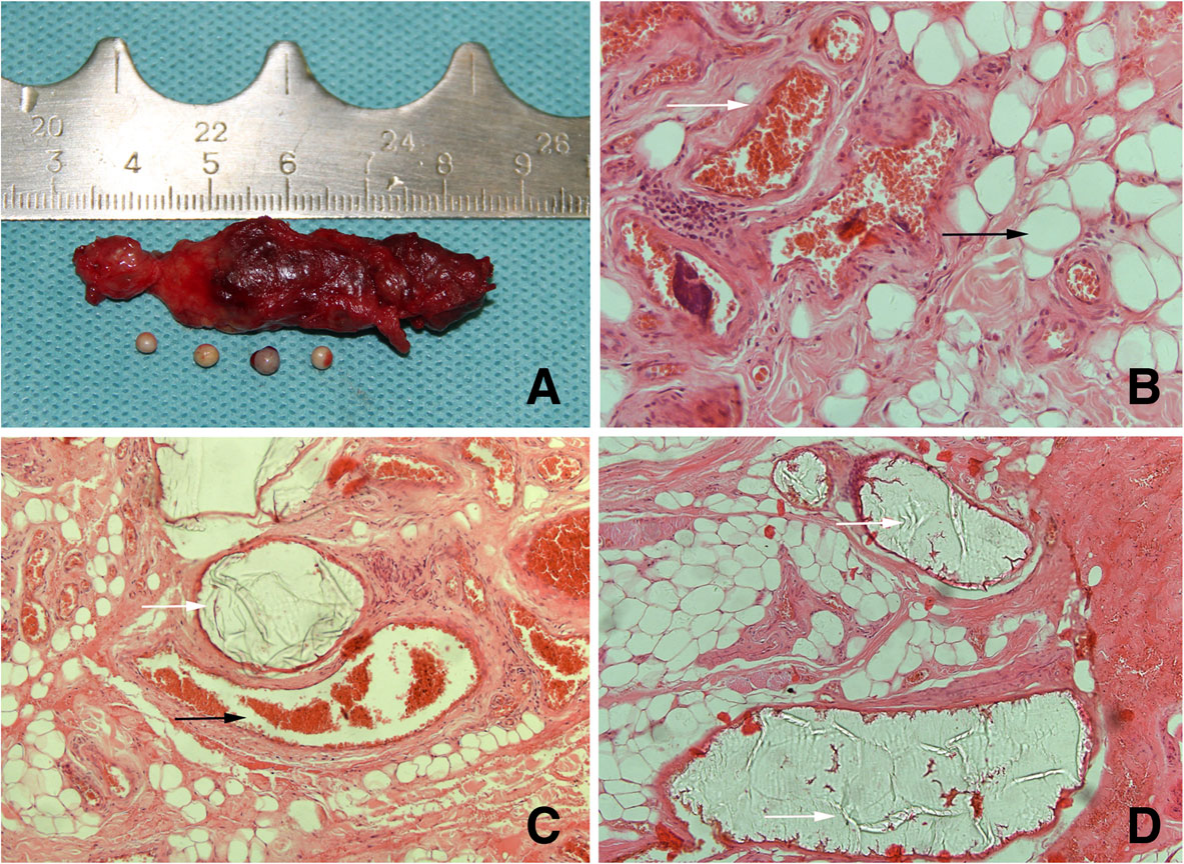
Histopathology examination of vascular malformations **a** Gross specimen of the venous malformation injected with medical glue was about 5.5 cm long and containing four phleboliths. **b** Microscopic examination of this vascular malformation tissue showed various blood vessel lumens of different sizes, the white arrow indicates the malformed blood vessel, and the black arrow indicates the fat cells inside the soft tissue. HE× 400 **c** White arrow indicates the transparent membrane material inside the vessel, namely the medical glue, while the black arrow indicates the malformed vein with many white blood cells inside the lumen instead of membranous material. **d** White arrow indicates the transparent membrane material inside the blood vessel. HE× 200

Pathological features showed in Table 3. Histopathological exam of the lesions injected with glue showed endovascular transparent membrane materials in routine sections stained with H&E. Most of the endothelial cells of the blood vessel were damaged, and only scarce of small vessel was present locally (Fig. 1).

**Table 3:** Pathological features of the cases

Pathological features	N
Intraosseous angioma	1
Arterial venous malformation	2
Venous malformation(varicosity included)	28 (5)

### Complication and prognosis

**Fig. 2:**
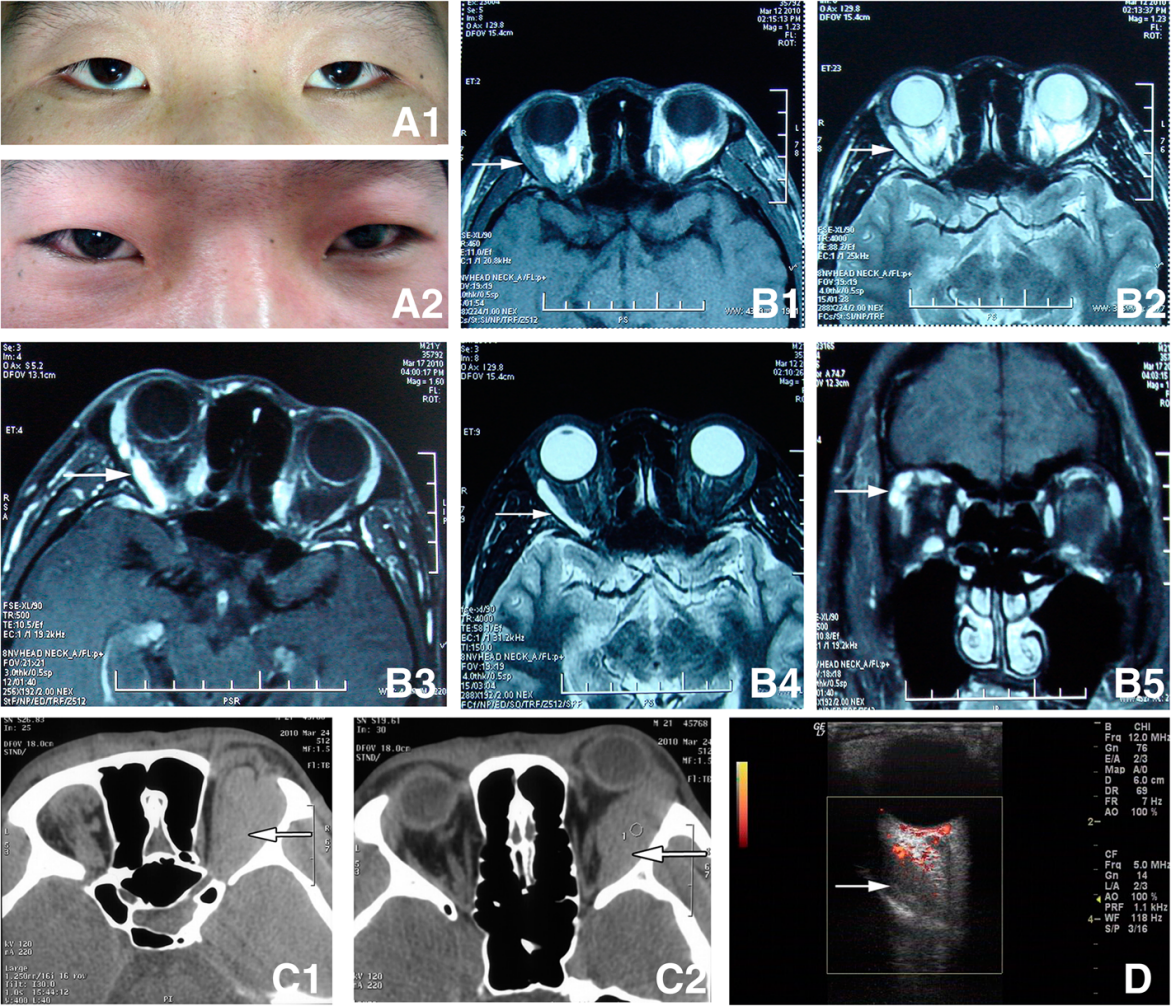
Patient with intra-orbital varicosity *A1*. Appearance when the patient was sitting, Hertel’s exophthalmometry revealed 2 mm of enophthalmos of right eye; *A2*. Appearance when the patient had bent down and bowed his head for 1 min, 4 mm of proptosis of right eye than the left; *B*. Horizontal MRI and coronal scan(supine position), showed a superotemporal ribbonlike mass in the extra-conal orbital compartment. The lesion displayed isointense on T1-weighted images (B1), hyperintense on T2-weighted images (B2), and significantly heterogenous contrast enhancement on enhanced and fat suppression MRI scan (B3–5). *C*. Axial CT scanning (prone position, the symbol of the right eye “R” was opposite to that on the MRI) showed a right superotemporal huge spindle-shaped homogenous mass pushing the eyeball to protrude and extending to the orbital apex. The superior rectus, lateral rectus and optic nerve were difficult to identify. *D*. CDFI (30 mmHg pressure was applied to the neck by the cuff of sphygmomanometer) showed a ribbonlike well defined hypoechoic mass expanded. The lesion was affecting the lateral rectus and pushed the optic nerve moving inward. There were signals of blood flow in the lesion. The arrow pointed to the lesion in Fig. 2

None of the cases had infection, complication of the cavernous sinus embolization, or necrosis of the local soft tissue (including application in the sub-conjunctiva lesions). In total, 15 cases had no complications. 9 cases had early post-surgical complications including mild ocular movement disorder. Follow-up of these cases showed that they all recovered gradually within three months. 2 cases had ptosis and recovered 1–2 months after the surgery. 2 cases had eyelid deformity and underwent plastic surgery one year later for cosmetic improvement. In addition, 2 cases had numbing of the skin, and 1 case had invagination of the eyeball. It is worth mentioning that one patient with intra-orbital varicosity had intermittent exophthalmos before surgery. Pre-operation imaging indicated that the malformed blood vessel was located in extraconal orbital compartment. When the malformed vessel dilated, it pushed the optic nerve displacement. During the surgery medical glue was applied and the lesion was resected. Because the lesion was large and deep into the orbital apex, a few glue residues at the orbit apex were not removed. Then a small tungsten wire wrapped by gelatin sponge was used as packing hemostasis. The size of the pupils was normal in post-operation. Pressure bandaging was applied as usual. The 3rd day after surgery vision monitoring showed uncertain light perception. Central retinal artery occlusion (CRAO) was confirmed after a promptly detailed ocular examination. Treatment for CRAO included ocular massage, administration of oxygen, sublingual nitroglycerin, and decreasing the intraocular pressure with intravenous mannitol. Patient condition improved with effective immediate rescue. Patient vision was 20/16 pre-operation, and 20/32 at the time of discharged (Fig. 2).

**Fig. 3:**
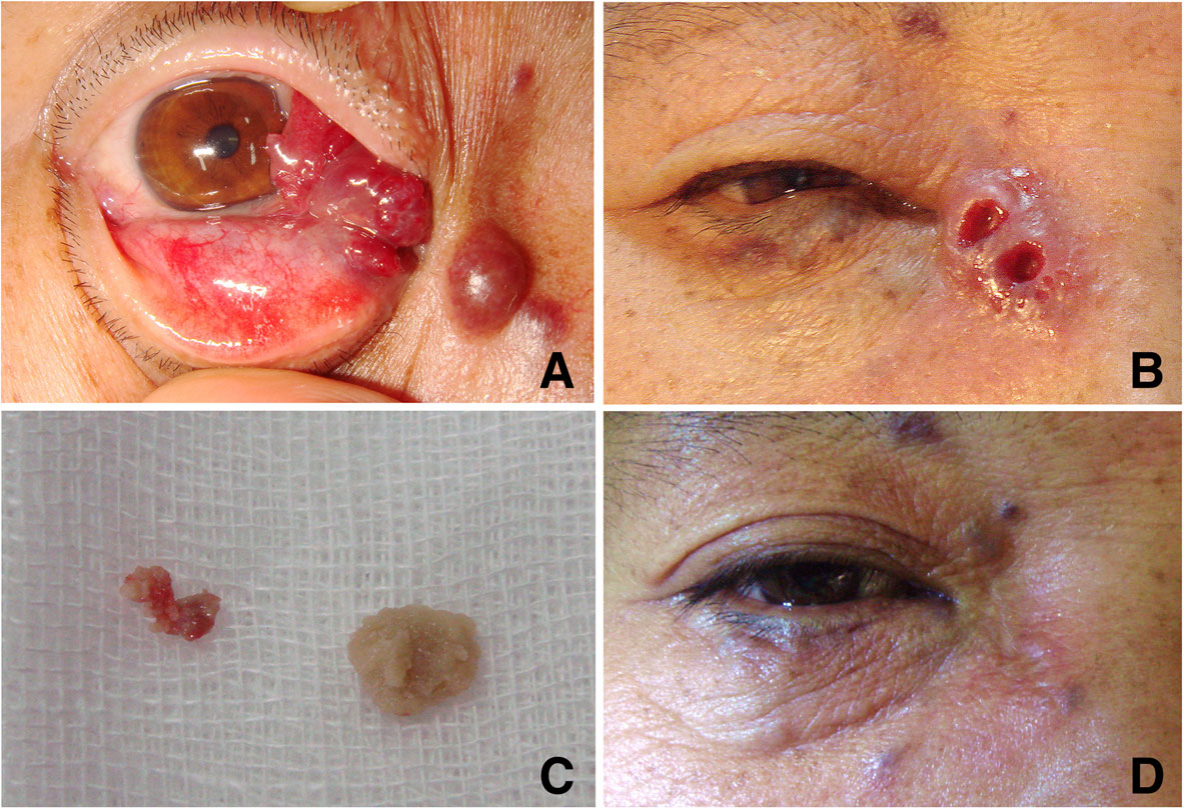
The 1st case with local skin adverse effect **a** Appearance before operation showed the purple obscure boundary lesion in the right nasal and inferior fornical conjunctiva and two spheroid masses under the skin of the inner canthus. Medical glue was injected into the two vascular malformation masses under the skin of the inner canthus via an incision made on the conjunctiva. The glue was not removed and the patient was discharged in stable condition one week after surgery. **b** The appearance of the lesion one month following the surgery showed two round skin ulcers on the nasal side, with 4 mm in diameter, and white swelling elevated margin. **c** Two residues of medical glue were removed from the ulcers; **d** local skin scar healing after debridement and suture

**Fig. 4:**
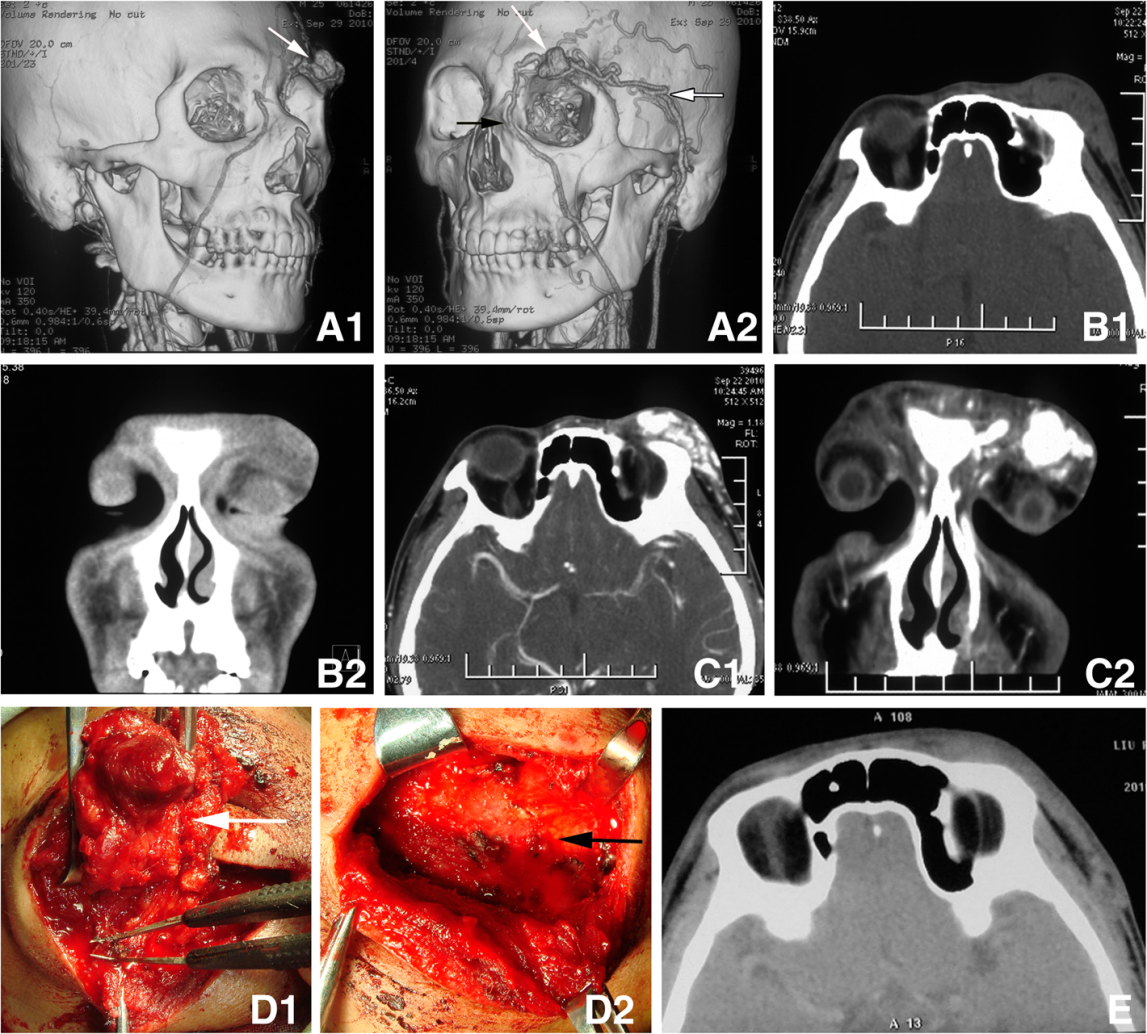
The 2nd case of local skin adverse effect *A* Three-dimensional computed tomographic(CT) angiography showed an abnormally expanded blood vessel mass (white arrow) in the soft tissue above the left orbit, supplied by the left superficial temporal artery, temporal artery (white arrow with dark outline) and arteria angularis (black arrow). The malformed blood vessel was not adjacent to the eyeball. *B*. CT imaging showed subcutaneous soft tissue mass in the upper left eyelid and forehead. *C*. Enhanced CT scan showed the vessel mass significantly heterogenous contrast enhancement. *D*. Surgical debulking was performed via a skin incision below the supraorbital ridge. White arrow indicates the solidified vascular malformation after medical glue injection. Black arrow indicates the exposed frontal bone after complete resection of the malformed blood vessel. *E*. CT scan showed the skin in the lateral-superior region of left orbit was slightly thickened at three months after surgery. Comparison of the pre-operation imaging the malformed blood vessel lesion was absent

**Fig. 5:**
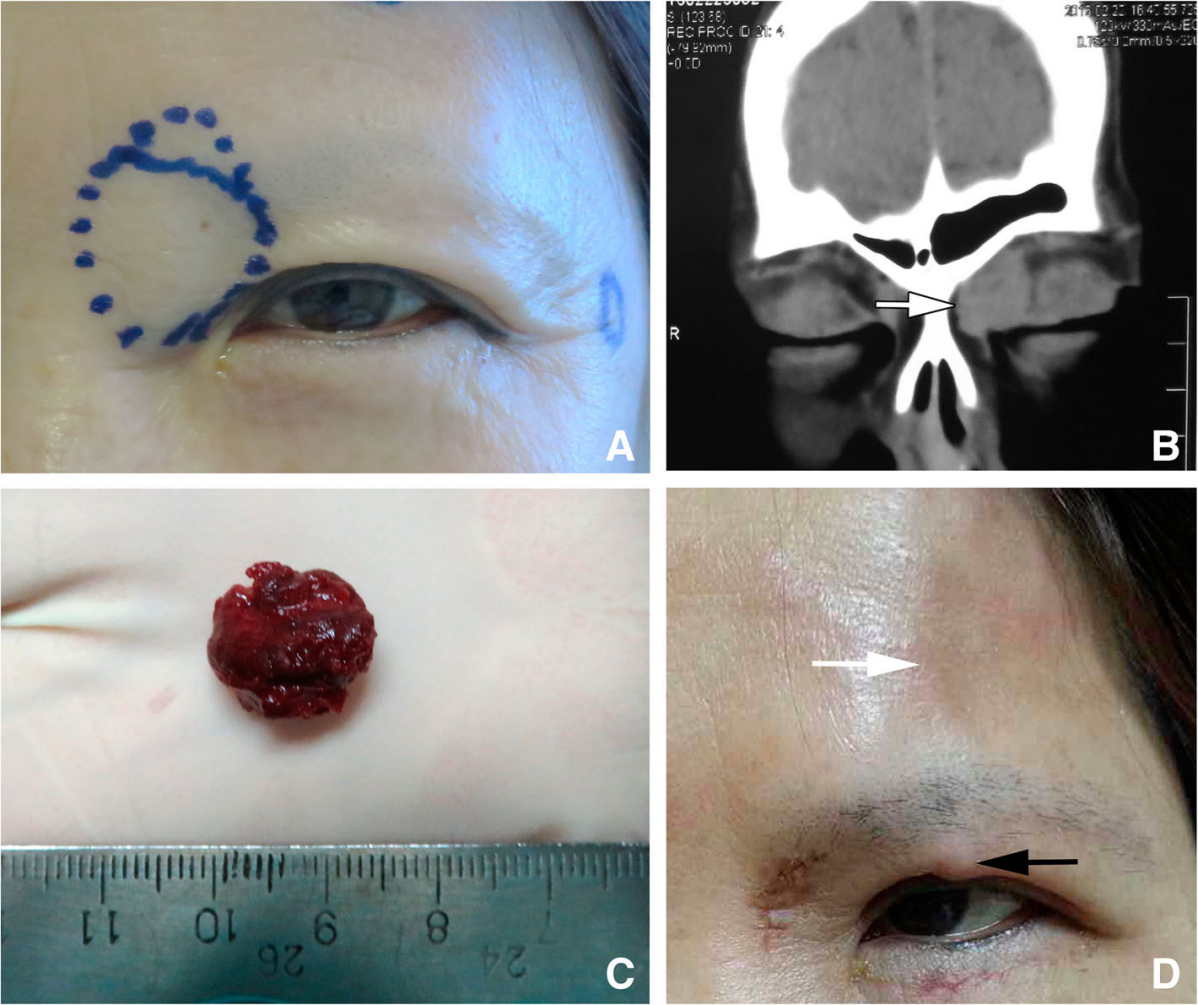
The 3rd case of local adverse effect to the skin. **a**. Appearance showed that the left nasal upper eyelid was slightly elevated. The border of the mass was drawn after putting pressure on the neck. **b**. CT coronal scanning showed an interior-superior homogenous mass in the left orbit (white arrow with dark outline). **c**. The gross specimen of vascular malformation solidified after medical glue injection. **d**. Follow-up two weeks after the surgery showed skin color change on the left forehead (white arrow), slightly congested, with an ill-defined border, and a hard nodule in the middle of the upper eyelid (black arrow)

There were 3 cases of adverse effects probably caused by medical glue, which all affected the injection side of the superficial eyelid lesions, and not limited to the surgical field. The 1st patient was treated in the early years. At that time, the injected medical glue was not removed. Regular follow-up in one month after the surgery showed round skin ulcer, and solidified medical glue as a foreign body was removed with tweezers. Immediate surgical exploration was performed to remove other medical glue, and the skin healed well in the end (Fig. 3). Another patient suffered from a mass lesion on the left upper eyelid and near the supraorbital ridge. The lesion was as big as a walnut and had ill-defined border, pulsatile, and audible wind-blowing murmur in the blood vessel. Computed tomography angiography (CTA) examination confirmed an abnormally expanded blood vessel mass above the left orbit, supplied by the left superficial temporal artery, temporal artery and arteria angularis. Pre-surgical examination showed the malformed blood vessels were adjacent to orbicularis oculi muscle and frontalis. During surgery the supplying blood vessels were ligated at first and then medical glue was injected into the lesion. The malformed blood vessels were treated in this way one by one. After removed all the malformed blood vessels, we carefully inspected the surgical field and cleaned every piece of medical glue to make sure that no glue residue before suturing of the incision. Pathological diagnosis showed arteriovenous malformation. When the suture removed at one week after the surgery, there was a palpable solid nodule with the size of a rice grain on the upper eyelid, and a 0.5 cm palpable strip of solid nodule under the temporal skin with mild congestion. Visual image is not available unfortunately. On the follow up visit 3 months after the surgery, the solid nodule had softened and the patient stopped following up (Fig. 4). In the third case of subcutaneous venous malformation, a suture was made after confirming that there was no residual glue. Two weeks after the surgery there was one solid nodule and mild congestion around but not in the surgical field. Nine months after the surgery during a phone call follow up, the nodule had softened but was still palpable, and the skin color was normal (Fig. 5).

Postoperative follow-up lasted for half a year to five years. A two-year-old patient had a recurrence shortly after the surgery. Because the vascular malformation was closed to the posterior pole of eyeball, the lesion was partially removed after embolization. Pathological report showed venous malformation. Four days after surgery the eyeball suddenly protruded after the patient cried and was immediately treated with surgery. The patient was followed up for 2 years and suffered from recurrent attack of intra-orbital hemorrhage. Another patient suffered from subconjunctival venous malformation far from the original surgical area (orbital apex) in the ipsilateral orbit and orbital venous malformation in the other orbit two and a half years following surgery. There are no recurrences in the other 29 cases.

## Discussion

The surgical resection of orbital vascular malformations includes a risk of hemorrhage, many complications, and even loss of vision in severe cases. Once a malformed blood vessels rupture during surgery, it is difficult to separate lesions from normal tissue, and may result in the incomplete resection of the lesion. Unfilled malformed blood vessels are even harder to remove completely. Hence, recurrence after surgery is common. Controlling for hypotension, tilting the patients head up, hemostatsis and preparations for blood transfusion are important measures needed to prevent and treat any intraoperative bleeding previously. In this article we reported on the application of medical glue embolization under direct surgical view, which rapidly molded and solidified in the malformed vessels with clearly defined borders, making it easier to resect the malformation completely and reduced blood loss.

In the past years, there have been many reports about the treatment of blood vessel malformation using embolization and many clinically available materials [1–6]. Medical adhesive is one such tool, which can be categorized according its application as a soft tissue glue, dental glue, bone cement or skin pressure sensitive adhesives. Soft tissue glue can be further subcategorized into bioadhesive and chemical adhesive. Fibrous protein glue, a bioadhesive, is widely used in ophthalmology, such as to glue the conjunctiva or amniotic membrane tissue together in pterygium excision and amniotic membrane transplantation [7]. The chemical adhesive α-cyanoacrylate has recently undergone rapid development, and is widely used in neurosurgery, general surgery and obstetrics and gynecological surgery [8–10]. The main gel content we used in this study was a highly purified α-Cyanoacrylic high carbon alkyl ester which polymerized instantly when mixed with weakly nucleophilicity materials (anionic materials including water, amidogen, alcohol, mild bases, and proteins or organic amine in organisms). The liquid medical gel can became solid adhesive medium to bind damaged tissue with solidification only taking about 6~15 s. It has a strong intensity, adhesiveness, and non-flowing features as well as strong antibiosis, bacteriostat activities. This is mainly applied to the treatment of cerebral spinal fluid leakage, cranioplasty and dura mater repair in neurosurgery. Its chemical features are stable; it does not degrade nor release toxic materials, and has good biological compatibility. Animal toxicity studies have demonstrated it is non-toxic, not carcinogenic nor teratogenic, and can be degraded or excreted, without toxicity accumulation [11]. The longer the molecular chain of the ester group, the less toxic is the ester [12]. The n-octyl ester is almost non-toxic. In clinical α-cyanoacrylate has been used as portal vein embolic agent for many years. It’s long-term effect is stable and there is less toxicity or adverse effects reported [13].

In ophthalmology, medical glue has been used to treat orbital burst fractures, orbital reconstruction, and restoration of enophthalmos deformity. It has proven to have good hemostasis and inner fixation [14]. Researchers used octyl-α-cyanoacrylate to treat corneosclera lacerations in an experimental animal model(Japanese white rabbits) in order to observe the tensile strength, wound healing and its biological compatibility with superficial eye tissues. This study confirmed that the glue was capable of rapid closing wound, and generating enough tensile strength to support wound healing. Glue has good biocompatibility and caused neither toxic nor pathological changes to the cornea and sclera. The time for wound healing and the tensile strength was normal. Its application could also prevent secondary injury during stitches removal. Using laser confocal microscopy to compare glue treatment with sutured cornea samples, it has been confirmed that medical glue suppressed the growth of new vessels and had no effect on wound healing time [15].

In our preliminary experimentation we found that medical glue immediately solidified once it came in contact with blood, thus obstructing continuous injection. We then inferred that different concentration of medical glue would have a different rate of solidification, so we attempted to dilute the glue with sterile WFI before injection. Unfortunately, the diluted medical glue became cloudy. We then stopped diluting the glue and used original glue for injection as part of clinical methodological improvement. We found several matters should be attended to during the application of glue surgical resection. ① The syringe used to draw/inject medical glue needs to be dry. Changing the needle after drawing glue can avoid the blockage caused by glue residue in the needle if it contact with blood when injected. ② The velocity of injection should be properly controlled. Slowly injection would cause the glue to solidify and block the syringe needle. Conversely, quickly injection might risk of injecting into the cavernous sinus. ③ The volume of injected medical glue is relevant to the range and size of the lesion. It’s better to appropriately inject the medical glue instead of it being overused. ④ When applying glue, the change in color and rigidity of the malformed blood vessels should be monitored closely in order to avoid glue leaking from the vessels. ⑤ Using an approach with multiple injection sites better guarantees procedure efficacy and safety.

There was one case of severe complication-- CRAO perioperative period in this case series. It is difficult to distinguish CRAO that may arise from the injection of medical glue or filling of tungsten wire, and other, more insidious reasons. As this was a preliminary trail with limited understanding of the medical glue, we used medical glue combined with tungsten wire to fill the region of vascular malformation for hemostasis. However, we are now convinced of the efficacy of medical glue embolization so we have stopped using tungsten wire filling method as it introduces a foreign body to inside human body, we. Some scholars reported that using medical glue for massive cerebral arterial-venous malformation, may be complicated by the embolization of draining vein or over-perfusion syndrome due to the difficulty in controlling the concentration and injection speed [8]. This suggests that clinicians should examine the safety of the intra-orbital application of medical glue for the embolization treatment.

Scholars have found that four months after using medical glue for brain tissue hemostasis, second surgery was performed due to tumor recurrence and residual glue were present intraoperatively. This finding was different from the complete absorption of glue observed in experimental animals [16]. Other scholars found that glue can cause neuronal necrosis, nerve fiber rupture and demyelination, and lead to T lymphocytes, mast cells and macrophages infiltration. It suggests that the application of medical glue to nervous tissue or other sensitive tissues must be carefully selected [17, 18]. In our early study, most patients did not have adverse reactions although medical glue was not completely removed from deep orbital lesions. However, there were local adverse reactions in three cases of superficial lesions of eyelid skin. One such case suffered from local inflammation and a skin ulcer due to residual glue in the subcutaneous vascular malformations. This residual glue was removed and patient was cured with a second operation. Further analysis of this case suggested that medical glue embolization affected local blood supply of the skin and induced local inflammatory response as a foreign body especially in the superficial lesions. In the two other cases, although intra-operative exploration did not find glue residue, the adverse skin reactions were still present after surgery, involving the skin of both the surgical area and the surrounding non-surgical area. It is speculated that the human body has individual differences in the inflammation response to medical glue. Some people with a sensitive constitution may have a rejection reaction. With the gradually recognition of medical glue, we made several improvements to treatment. ① Before surgery, patients were informed of the advantages of using medical glue, and the possible adverse effects of local inflammatory response or even rejection caused by medical glue and these adverse effects may have individual differences. All patients gave informed consent. ② During the intra-operative injection, we ensured that the glues did not penetrate deformed blood vessels, and do not overflow from the puncture site. ③ For abnormal blood vessels with a rich blood supply, such as varicosity and arterial-venous malformations, ligation of the blood supply vessels at first during the surgery (including the draining vein and the blood supplying artery) may keep the medical glue from spreading and the embolization of other tissues. ④ In view of the existing basic research and clinical observation results, it is recommended that the medical glue be considered as a foreign body, with cautious and careful application. It should be removed as much as possible after application to avoid residue. Medical glue in the orbital apex should be evaluated the relationship between the optic nerve and/or the eyeball in order to decide whether to remove it.

In light of the above analysis we believe that it is feasible to use intra-vascular injection of medical glue for the embolization of orbital vascular malformation. However, since there is a different growth pattern of deformed blood vessels (isolated or diffuse), a different growth range, and a different blood flow velocity within the lumen, they all affect the dispersion rate of medical glue in blood vessels. Hence, injecting the same volume of glue may lead to a different range of solidification. At present, the quantitative relationship between the amount of medical glue injected and the volume of the lesion has not been established. At the same time, considering the adverse reactions of medical glue, we recommend that re-injecting according to the scope of solidified vascular malformation and the lesion size is not an absolute indication. Our key finding and clinical recommendations were, first when applying medical glue to superficially located vascular malformations, attention should be paid to avoid leaving glue residues; all glue should be removed to minimize complications. Next, when applying medical glue into a deeply located orbital vascular malformation, be sure to protect the surrounding normal structure and to ensure “intratumoral injection.” Additionally, when applying medical glue to the vascular mass closely located near the optic nerve or eyeball wall, attention should be paid to the amount of glue used. It can be used for a small amount and multiple times, to avoid its impact on eye blood supply. Finally, lesions rich in blood flow and affecting a large area, it is recommended to first ligate the supplying blood vessels, manage the mass separately, and then inject glue for embolization.

## Conclusion

Orbital and eyelid vascular malformation is a very complicated illness, which is difficult to manage, so an indication for surgical resection should be critically evaluated. The application of medical glue can reduce the risk of intra- and post-operative hemorrhage, as well as may provide a ‘bloodless’ surgical field for surgeons. After molding and solidification using medical glue, malformed blood vessels are easy to remove. Such application of glue facilitates the complete resection of ill-defined lesion and reduces post-surgical recurrence. Its application is not simply a local injection and thus requires extensive experience in orbital surgery and techniques, as well as adequate preparation for the timely management of sudden onset complications.
